# Atypical Presentation of Kimura's Disease in a Male Patient: A Case Report and Review of Literature

**DOI:** 10.1155/2022/5103547

**Published:** 2022-12-06

**Authors:** Zahra A. Natsha, Islam A. Hamarsheh, Rawan S. Utt, Bassam Abu Alrob, Adnan A. Wahdan

**Affiliations:** ^1^Al-Quds University Faculty of Medicine, Jerusalem, State of Palestine; ^2^Palestine Medical Complex, Ramallah, State of Palestine

## Abstract

Kimura's disease (KD) is a rare chronic inflammatory condition of unknown aetiology. It is a benign disease that might mimic a neoplastic process. It primarily affects the head and neck region, presenting as deep subcutaneous masses, and is often accompanied by triad regional lymphadenopathy, salivary gland involvement, and high serum immunoglobulin *E* (IgE) levels. Here, we report the second documented case of KD in Palestine diagnosed in a 28-year-old male patient who presented with lymphadenopathy and increased serum immunoglobulin *E* and *G* (IgE and IgG) associated with intermittent abdominal pain, generalised fatigue, hepatomegaly, cardiomyopathy, reactive airway disease, peripheral vasculopathy, peripheral neuropathy, and focal segmental glomerulosclerosis. The patient was managed with steroids and an immunosuppressant (Azathioprine) with a moderate response for two years. In 2021, treatment with Mycophenolate Mofetil was initiated, which was more effective than Azathioprine.

## 1. Introduction

Kimura's disease (KD) is a rare chronic inflammatory disorder with angiolymphatic proliferation [1], seen more commonly in Asian people [2] and most people aged between 20 and 40 years old [3]. Males are affected more commonly than females, with a ratio of 3.5–7:1 [4]. Typically, it presents as a subacute painless mass in the head and neck region, accompanied by regional lymphadenopathy [5]. It may be associated with eosinophilia and elevated serum immunoglobulin *E* levels [1]. The diagnosis depends on the mass biopsy or excision for histopathological and immunohistochemistry evaluation [3].

There are different modalities for treatment, including surgical resection, radiotherapy, immunotherapy, and oral corticosteroids, but the best choice is still controversial [6, 7]. However, one case was reported with spontaneous resolution [4]. The prognosis is good, and the incidence of associated renal disease is between 10% and 60% [8], but less than 10% develop nephrotic syndrome (NS) and more complicated courses [6].

## 2. Case Presentation

A 28-year-old male patient, a heavy smoker with a body mass index of 24.8, with a history of hepatitis A 3 months before admission, and asthma since the age of 19, was admitted with one week of colicky periumbilical pain radiating to the lower abdomen and upper thighs. The pain gradually increased in intensity and was associated with distension, general fatigue, a fever of 39.1°C, anorexia, and unintentional weight loss (around 10 kg in one month). Also, he reported shortness of breath, lower limb swelling, and upper back and bilateral hand numbness that progressed to severe pain. There were no reports of trauma, heartburn, vomiting, diarrhoea, flatulence, melena, or haematochezia.

The patient was admitted to the intensive care unit at the age of 19 with exertional dyspnoea, orthopnoea, and lower limb oedema, according to his medical history. A diagnosis of pneumonia with atelectasis and delirium was made. He also had a history of diffuse nasal polyposis, grade 4, as well as an allergy to nonsteroidal anti-inflammatory drugs (NSAIDs) and penicillin. Additionally, at the age of 2, he underwent adenoidectomy and tonsillectomy procedures.

**Figure 1:**
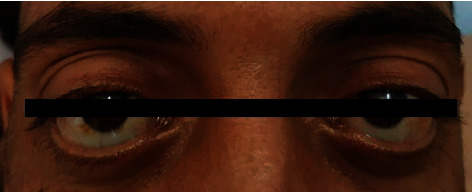
Marked bilateral exophthalmos.

**Figure 2:**
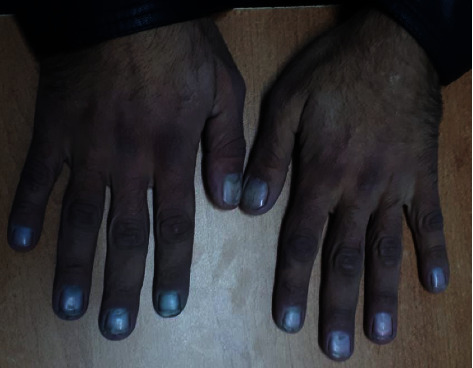
Bilateral Raynaud's hands, with marked bluish discoloration of the fingers and nails.

Chest examination revealed barrel shape, diminished expansion and faint wheezes. Additionally, hepatomegaly with a 20 cm liver span in the midclavicular line was found and confirmed by an ultrasound study. Both inguinal and cervical lymph nodes were palpable, bilaterally firm, rounded, nontender, and 1 to 2 cm each in size. Over the abdomen, audible bowel gurgling sounds were heard. Moreover, marked bilateral exophthalmos (Figure 1) and bilateral Raynaud's hands with bluish discolouration were observed (Figure 2). His right radial pulse was absent, whereas the left radial pulse was weak. On the other hand, the examination of the lower limbs was normal, and no discrepancies between arm and leg blood pressure were found.

Laboratory tests revealed anaemia and elevated levels of IgE and IgG, inflammatory markers, and the creatinine level. In addition, rheumatoid factor and antismooth muscle antibodies were positive (Table 1). However, electrolyte levels, liver enzymes, a thyroid function test, serological analysis, thrombophilia studies, an albumin blood test, and tumour biomarkers (PSA, CEA, and AFP) were all normal.

A computed tomography (CT) Pan-Scan showed mild bronchial wall thickening and centrilobular opacification. Hepatomegaly with an inhomogeneous mottled pattern of contrast enhancement was seen. Para-aortic, mediastinal, and hilar LNs were small, none exceeding 6 mm multiple small inguinal LNs were noted, none over 12 mm. Sinus CT without contrast revealed a paranasal sinus mucocele.

Three months after admission, laboratory tests showed 3+ proteinuria and 1.2 mg/dL creatinine. An echocardiogram revealed dilated left ventricle (6 cm), reduced systolic function, mild left ventricular hypertrophy, mildly dilated chambers, and moderate aortic regurgitation (interventricular septum thickness 1.2 cm, EF = 40%). As a result, heart failure therapy (enalapril, spironolactone, and carvedilol) was initiated.

An inguinal LN biopsy showed lymphoid follicles an increased number of eosinophils surrounding dilated and hyalinised blood vessels. No evidence of malignancy was found. Features were mostly consistent with Kimura's disease. A biopsy of the bone marrow revealed normocellular bone marrow with a granulomatous reaction and eosinophilic infiltration. The kidney biopsy revealed glomerular segmental sclerosis, and a diagnosis of focal segmental glomerulosclerosis was made. Therefore, moderate-dose steroids (tapered within a few weeks) and Azathioprine were added to his medications regimen.

**Figure 3:**
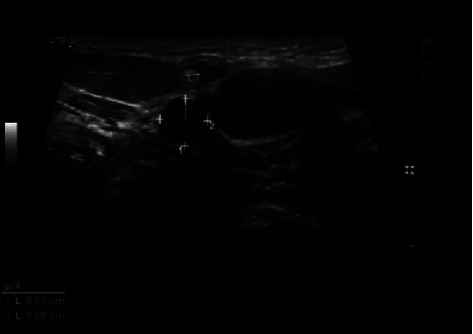
Neck ultrasound of the left lateral aspect of the neck shows a 7 × 7 mm rounded lymph node, with loss of fatty hilum, located medially to the internal jugular vein.

**Figure 4:**
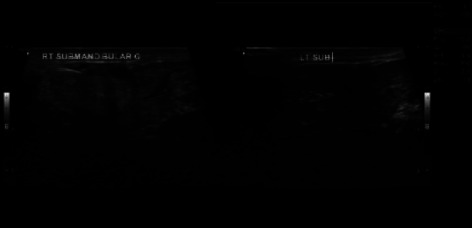
Neck ultrasound of both submandibular glands which shows left submandibular gland coarse echotexture, normal size, and vascularity.

**Figure 5:**
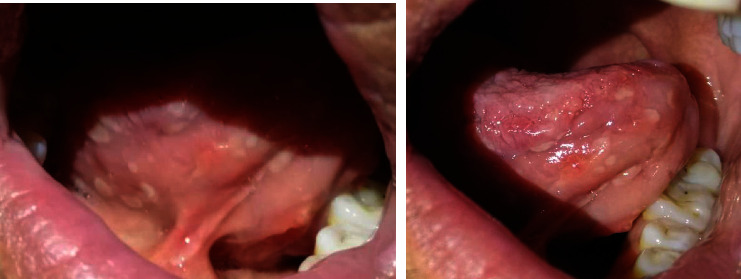
Ventral white tongue ulcers.

Contrary to medical advice, he has been off medication throughout 2019. He developed a right round, firm, and nontender lump in his neck. The US revealed approximately 7 × 7 mm LN, located in the middle of the left lateral aspect of the neck with loss of the hilum, and a left submandibular gland of coarse echotexture (Figures 3 and 4). He also experienced paraesthesia in the right arm and right leg; electromyography indicated right tibial nerve axonal neuropathy. Moreover, he had several white, well-defined, and painless tongue ulcers (Figure 5) that were persistent over a year, oval in shape, less than 1 cm in size, more than 14 in number, and distributed along the anteromedial and the anterolateral portions of the ventral aspect of the tongue.

A year later, Mycophenolate Mofetil was started in place of Azathioprine since it was more effective in suppressing the disease and reducing GI distress. The patient's most recent examination revealed improved symptoms of asthma and cardiomyopathy. His last echo revealed normal LV size and function (nearly 55% ejection fraction), and serum IgG and IgE levels were within the normal range.

## 3. Discussion

This is the second case of KD reported in Palestine. The patient manifested rare and atypical symptoms as he developed multiple systemic manifestations, including renal, cardiac, respiratory, gastrointestinal, and neurological involvement. KD is a benign chronic inflammatory rare disease that is more common in males and Asians. It usually presents as subcutaneous nodules at the head and neck region, and lymph node enlargement. Patients with KD usually have high IgE levels and eosinophilia, which suggests shared pathological and genetic pathways with other immunological diseases such as asthma [9], which this patient also has. However, the combination of systemic symptoms observed in this case is typically absent in KD.

Several factors were suggested to have a role in the pathogenesis of KD, including neoplasms, insect bites, candida infection, immune dysregulation, and genetic risk factors [10, 11]. Ohta et al. indicated that the levels of Th2 and Tc1 cells were higher in KD patients compared to controls [12]. The overproduction of Th2 cytokines, such as granulocyte-macrophage colony-stimulating factor (GM-CSF), tumour necrosis factor-alpha (TNF-*α*), interleukins (IL-4, IL-5, and IL-13), eotaxin, and RANTES (Regulated on Activation, Normal *T* Cell Expressed, and Secreted), trigger the production of the lymphoid follicle and high IgE [12, 13]. Therefore, immunohistochemistry is essential for diagnosis and excluding alternative diagnoses.

Many KD patients also suffer from asthma, rhinitis, eczema, and chronic urticaria, which suggest shared immunological pathogenesis [14]. Liu et al. indicated that patients with KD may have a genetic predisposition towards allergies. Persistent or interrupted exposure to a known or unknown allergen could trigger the occurrence of KD by the antigen-presenting cells that promote the differentiation of CD4+ T cells into Th2 cells during T cell priming. Thus, elevated IgE and abundant eosinophils with mast cells and basophils result in type 1 hypersensitivity [7].

Using the OMIM and GeneCards databases, Chen et al. found that genes linked to autoimmune diseases, such as ARPC1B, NOD2, BANK1, and CIITA, may be related to the susceptibility to KD [11]. Volpi et al. analysed 14 patients with biallelic ARPC1B mutations and reported combined immunodeficiency, allergy, and autoinflammation as a result of the mutations. Clinical symptoms reported in these patients involved platelet abnormalities, eosinophilia, immune-mediated inflammatory diseases, and combined immunodeficiency. Moreover, abnormal immunoglobulin levels were observed in vivo, with markedly elevated IgA and IgE in almost all cases [15], suggesting that ARPC1B mutations are associated with immunologic derangement or autoimmune disease, which may be associated with a predisposition to KD. However, genetic mutations associated with KD susceptibility remain unclear, and further investigations are required [11].

Chen et al. compiled that patients with systemic lupus erythematosus (SLE), Behcet's disease (BD), ankylosing spondylitis (AS), minimal change disease, membranous nephropathy (MN), mesangioproliferative disease, IgA nephropathy, and IgG4-related diseases (IgG4-RD) have been reported to have KD [11, 16]. Moreover, they highlighted the numerous reports of the co-occurrence of KD and IgG4-RD, which demonstrates that KD is more prevalent in individuals with autoimmune or autoinflammatory diseases [11].

Therefore, histopathological and immunohistochemistry evaluations are necessary to distinguish KD from other similar conditions. Typical histological features of Kimura's disease in lymph nodes include germinal centre hyperplasia, increased postcapillary venules in the mantle zones, vascularization of germinal centres, eosinophilic infiltration in the sinuses and paracortex occurring later in germinal centres, paracortical eosinophilic abscesses, and sclerosis of variable degrees [7]. The pathological findings must be interpreted in combination with the clinical, serologic, and radiographic data.

It is important to rule out other inflammatory and malignant diseases with similar clinical presentation and pathological features. KD and Hodgkin Lymphoma, for instance, share pathological characteristics, including intact LN architecture, florid germinal centre hyperplasia, extensive eosinophilic infiltrates, and proliferation of postcapillary venules. Even though the presence of Reed-Sternberg is considered a diagnosis for Hodgkin Lymphoma, it may not be detected initially, which makes a diagnostic challenge [17].

The differential diagnosis of KD is quite broad, including IgG4-RD, angiolymphoid hyperplasia with eosinophilia (ALHE), Langerhans cell histiocytosis, Churg-Strauss syndrome, Castleman's disease, lymphadenopathy of drug reaction, parasitic lymphadenopathy, and others [17]. The top on the differentials list is ALHE disease. Clinically, both diseases have soft tissue masses, especially in the head and neck region, with a prolonged clinical course and tendency to recur. They share a similar vascular nature of the lesion with lymphoid and eosinophilic infiltrates, and both have a good prognosis [18, 19].

But there are some differences: KD usually affects younger people in the second or third decades, more commonly in males and Asian people, and is almost always accompanied by peripheral blood eosinophilia and elevated serum IgE levels [5, 19]. Our case showed an elevated IgE level (more than 4000). On the other hand, ALHE appears in all ages, with a peak incidence in patients who are 30–50 years old (mean age of 38 years); 6% of cases appear in children, and there is no gender predilection. It has been reported most frequently and similarly in Asians and whites, with less than 5% of reported cases in blacks [20, 21]. ALHE causes cutaneous papules and nodules and is less commonly associated with eosinophilia [22].

In addition, KD and ALHE manifestations on the tongue and the oral mucosa are rare. In the latter, tongue ulcers are approximately 10 mm in diameter, while KD oral ulcers tend to be larger, about 30 mm [23, 24]. In our case, the patient developed multiple ventral tongue ulcers that persisted for over a year, which could be a manifestation of KD.

However, KD and ALHE can rarely affect the orbit and the ocular adnexa. They can cause proptosis, lid swelling, ocular dysmotility, or a palpable mass [25, 26]. In our case, the patient presented with bilateral exophthalmos. Moreover, the involvement of an orbital pseudotumor, proptosis, lacrimal gland, and trigeminal nerve had been reported with IgG4-RD, which shares similar features with KD [27].

Liu et al. indicated that eosinophilia or high IgE levels and lymphadenopathy are often found in patients with IgG4-RD and KD. Similarly, IgG4-RD is an immune-mediated fibroinflammatory condition. It has systemic manifestations characterised by the extensive infiltration of IgG4-positive plasma cells and T lymphocytes into several organs, such as the pancreas, lung, kidney, salivary gland, lymph nodes, gallbladder, bile duct, retroperitoneum, prostate, and ocular adnexa. It has been reported in the literature that pathologically induced tissue fibrosis with obliterative phlebitis and vasculitis was noted in IgG4-RD. Immunoglobulin class switching of B cells to IgG4 and IgE depends on IL-4/IL-13. Indeed, because antigens or abnormal immune factors that induce IgE and eosinophil responses are good inducers of IgG4, immunohistochemistry of IgG4-and IgG-positive plasma cells, and detection of IL-4, IL-5, IL-10, IL-13, and TGF-*β* should be considered to investigate better and understand the relationship between KD and IgG4-RD [7, 20]. Further immunohistochemistry investigations were not performed for our patient, but the clinical manifestations and the lymph node histopathology reports were enough to distinguish KD from IgG-4 RD. However, the increase in his blood IgG levels could be due to KD.

Lee et al. explained that IgG4-related lymphadenopathy exhibits a wide morphological variety that shows five different histological patterns. The KD pattern resembles type number five, which is the least common. It is characterised by focal replacement of the nodal parenchyma by scleral-hyaline tissue with variable infiltration of plasma cells, lymphocytes and eosinophils. Lee and colleagues also highlighted that several morphological features could be used to distinguish KD from IgG4-RD, despite the similarities in lymph node involvement between KD and variant five of IgG4-RD. However, they suggested that the presence of any IgG4-positive plasma cells in the peripheral blood as well as infiltration of IgG4-positive plasma cells in lymph nodes and skin lesions is all epiphenomena of Kimura's disease [20].

Kottler et al. reported a similar clinical pathology pattern of skin lesions in 25 KD patients to cutaneous IgG4-RD. They also compared 22 KD cases with skin lesions versus 27 published cases of cutaneous IgG4-RD and concluded that both conditions seem to be distinct entities based on clinical and pathological differences. For example, the median age of KD patients is 44.5, whereas the median age of IgG4-RD patients is 63. In addition, no patient with KD has been reported to have pancreatic involvement, whereas autoimmune pancreatitis is a distinguishing feature of IgG4-RD [23].

Moreover, pathological differences in nodal KD and IgG4-related lymphadenopathy have been described in the literature. For instance, the expression of IgE in follicular dendritic cells and eosinophilic microabscesses is only found in KD. Kottler et al. emphasises that clinical features are crucial to differentiate between both conditions [23, 27]. Indeed, KD shares many physiopathological mechanisms with several diseases, and further research into the aetiology is required.

Surgical excision, radiotherapy, surgical resection combined with low-dose postoperative radiotherapy, and oral corticosteroids are some of the available treatment modalities, but the optimal method is still controversial. Radiotherapy is preferred in cases of recurrent KD when other treatments have failed to control the disease [21, 25]. Some patients had a positive response to other immunosuppressants, such as Mycophenolate Mofetil [22, 27]. Our patient improved when Mycophenolate Mofetil was used, while his previous treatment with Azathioprine was less effective. However, due to the limited number of cases and the lack of randomised trials, the efficacy of therapy has not been adequately studied.

According to Ye et al. surgical resection followed by postoperative low-dose radiotherapy can achieve the lowest recurrence rate [6]. Senel et al. [13] used immunosuppressants (steroids, Azathioprine, and Cyclosporine) to treat KD patients with focal segmental glomerulosclerosis (FSGS). They were able to achieve quick improvement, and this regimen was recommended for cases with extensive fibrosis [24]. Romao et al. used corticosteroid monotherapy for a patient with FSGS, but the kidney function deterioration was rapid and ended with a kidney transplant [28]. In our case, the patient was treated with steroids and immunosuppressants (Azathioprine) after a diagnosis of FSGS was made.

## 4. Conclusions

Despite the unclear presentation and aetiology, Kimura's disease should be considered in the differential diagnosis of a subcutaneous neck mass and multiple subcutaneous nodules, especially when the condition appears with atypical systemic involvement and similarity to other hypereosinophilic conditions and IGg-4-related diseases. Indeed, the diagnosis should be based on the combination of characteristic histopathologic, clinical, serologic, and radiologic findings. To ensure favourable outcomes and prevent renal and thrombotic complications, treatment should aim to preserve the function and appearance of affected tissues.

## Figures and Tables

**Table 1:** Laboratory tests that were conducted as a part of the investigation.

Tests	Results	Reference ranges
Haemoglobin level (Hb)	11.5 g/dL	(13.5–17.5 g/dL)
Haematocrit (HCT)	36%	(43.5–53.7%)
Mean corpuscular volume (MCV)	78.3 fL	(80–100 fL)
Mean corpuscular haemoglobin (MCH)	25 pg	(27–31.2 pg)
Total white blood count was (WBC)	14.3 × 10^9^/L	0-
Total bilirubin	1.255 mg/dL	(0–1.0 mg/dL)
First-hour erythrocyte sedimentation rate (ESR)	40 mm/hr	(0–15 mm/hr)
C-reactive protein (CRP)	60 mg/dL	Below 3.0 mg/dL
Alkaline phosphatase (ALP)	260 IU/L	(44–147 IU/L)
Creatinine	1.37 mg/dL	(0.7–1.2 mg/dL)
Alpha 1 antitrypsin	43 mg/dL	(80–200 mg/dL)
IgG level	2126 g/L	(0–16 g/L)
IgE level	Over 4000 IU/ml	(150–1000 IU/ml)
Rheumatoid factor (RF)	Positive	—
Antismooth muscle antibody (ASMA).	Positive	—
Extractable nuclear antigen (ENA)	Negative	—
Antidouble stranded DNA (anti-dsDNA)	Negative	—
Antineutrophil cytoplasmic antibodies (ANCA)	Negative	—
Antinuclear antibody (ANA)	Negative	—

## Data Availability

The radiographic data and laboratory tests used to support the findings of this study are included within the article. Any other data related to this research are available from the corresponding author on reasonable request.
